# Supplementary material for: Analysis of PPARγ Signaling Activity in Psoriasis
Source: Int J Mol Sci. 2021 Aug 10;22(16):8603. doi: 10.3390/ijms22168603 (PMC8395241; doi:10.3390/ijms22168603)
Supplement: Supplementary file 1 [file ijms-22-08603-s001.zip › Supplemental materials_Analysis of PPARg signaling activity in psoriasis/Pathway models/Models images and html files/PPARG inhibits phenotypes assotiated with psoriasis/142924.html]

neutrophil migration


# Cell Process neutrophil migration

|  |  |
| --- | --- |
| URN | urn:agi-cellproc:neutrophil%20migration |
| Total Entities | 1 |
| Connectivity | 1919 |
| Name | neutrophil migration |

---

|  |  |
| --- | --- |
| ChildConcepts | neutrophil extravasation |

---

|  |  |
| --- | --- |
| Pathway | Keep out Signal: LTF Inhibits Neutrophil Recruitment and Inflammation |
|  | N2 Neutrophils in Tumor-Promoting Inflammation and Tumor Progression |
|  | PPARG inhibits phenotypes assotiated with psoriasis |
|  | efects selected |

---

|  |  |
| --- | --- |
| MedScan ID | 4000804 |

---

|  |  |
| --- | --- |
| Alias | neutrophilic transmigration |
|  | neutrophil transmigration |
|  | migration of neutrophil |
|  | migration by neutrophils |
|  | migration of neutrophils |
|  | neutrophilic granulocyte migration |
|  | neutrophil migration |
|  | neutrophils migration |
|  | neutrophilic migration |

---

|  |  |
| --- | --- |
| CellType | neutrophil {CellType urn:agi-ncimcelltype:C0027950} |
|  | neutrophil |

---

|  |  |
| --- | --- |
| CellProcess | cell migration {CellProcess urn:agi-gocellproc:0016477} |

---
